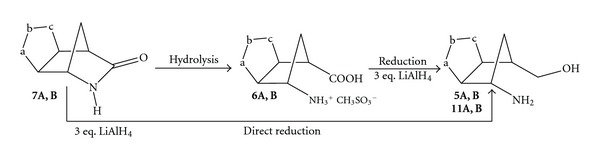# The Chemoselective Reduction of Isoxazoline **γ**-Lactams Through Iminium Aza-Diels-Alder Reactions: A Short-Cut Synthesis of Aminols as Valuable Intermediates towards Nucleoside Derivatives

**DOI:** 10.1100/2012/643647

**Published:** 2012-05-02

**Authors:** Misal Giuseppe Memeo, Mariella Mella, Paolo Quadrelli

**Affiliations:** Dipartimento di Chimica, Università degli Studi di Pavia, Viale Taramelli 12, 27100 Pavia, Italy

## Abstract

Isoxazoline **γ**-lactams are prepared starting from the regioisomeric cycloadducts of benzonitrile oxide to the *N*-alkyl 2-azanorbornenes taking advantage of the efficient catalytic oxidation by RuO_4_. The reduction of the amide groups is easily conducted in the presence of LiAlH_4_ under mild conditions, which allowed for the chemoselective reduction of the amide moiety followed by ring opening to afford the desired conformationally locked isoxazoline-carbocyclic aminols, as valuable intermediates for nucleoside synthesis.

## 1. Introduction

Amino alcohols or aminols represent a class of relevant compounds, whose utility in organic synthesis is testified by their use as key building blocks and by the wide variety of biologically active compounds prepared using these stable intermediates [1–4]. The importance of vicinal aminols is also well recognized in asymmetric synthesis and many chiral auxiliaries and ligands contain these substructures [5, 6]. The insertion of an amino and an hydroxy functionalities in a stereo ordinate way often determines the relevance and goodness of a synthetic method [7–13].

**Scheme 1:**
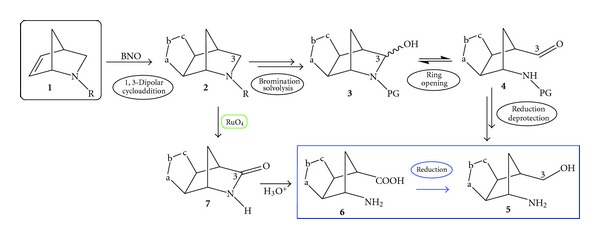


Recently, we have proposed a novel approach to useful aminols for the synthesis of carbocyclic nucleosides starting from an extremely convenient source, the 2-azanorborn-5-ene **1** (Scheme 1) [14], prepared by Grieco from the cycloaddition of cyclopentadiene with iminium salts generated *in situ* under Mannich-like conditions, in a mild and “green” aqueous aza-Diels-Alder (ADA) reaction [15–26]. The first iminium salts as dienophiles in DA reactions were used by Böhme et al. for the synthesis of piperidinium salts [27] in 1963 but there is no doubt that the appearance of the first paper by Larsen and Grieco in 1985 determined the success in the use of unactivated iminium salts as dienophiles to prepare ADA cycloadducts in very good yields [18]. Despite the easy availability of these hetero-cycloadducts, no attempts have been made for their use in nucleosidic syntheses in general, or, in detail, in order to prepare conveniently the lactam derivatives as useful key intermediates towards aminols, the direct precursors of nucleoside structures. In order to apply this strategy, the aza-methylene bridge has to be modified, and the carbon C3 must be oxidized by appropriate procedures able to unmask the amide functionality [14]. The stable 2-azanorbornene of type **1** (Scheme 1, R=CH_2_-Ph) displays a moderate dipolarophilic activity towards benzonitrile oxide (BNO) and the 1,3-dipolar cycloaddition reaction affords the regioisomeric cycloadducts of type **2**. Compared with norbornene, *N*-benzyl-2-azanorbornene still remains a highly reactive dipolarophile but less than the classical BNO trapping agent that is twice more reactive in apolar solvents and three times in polar or polarizable solvents [14].

Then, we transformed the cycloadducts **2** into more convenient derivatives, through the oxidation to the corresponding *N*-oxides and their conversion into *N*-acetyl derivatives via the mild Polonovski rearrangement [28, 29]. Insertion of an acetyl as an amine protecting group (PG) on the 1,3-dipolar cycloadducts and creation of an amide group nearby the methylene in position 3 allowed for the oxidation at the carbon atom C3 by means of NBS/AIBN bromination reaction, which afforded a complex mixture of products whose elaboration allowed for the isolation of the protected aldehyde **4** and ring opening. Reduction of the aldehyde group and deprotection afforded the target aminols **5**. The overall protocol required from the beginning 8 steps to prepare the aminols **5**, which were further converted into the desired adenine nucleoside derivatives [30, 31], which were submitted for biological tests against a variety of viruses.

In the search for a fast and convenient oxidation protocol we took advantage of the oxidative ability of RuO_4_, a strong oxidizing agent. Since its introduction into organic chemistry more than fifty years ago [32], RuO_4_-catalyzed reactions in a biphasic system were often considered to be sluggish or unselective [33]. Two recent papers by Petride and coworkers [34, 35] dealing with the RuO_4_-mediated oxidation of *N*-benzylated tertiary amines (cyclic and acyclic) prompted us to test the RuO_4_ on our tricyclic isoxazolino-2-azanorbornene derivatives of type **2** probing the regioselectivity of the oxidation process without replacing the benzyl group at the nitrogen atom or any other structure modification activating the adjacent methylene towards oxidation. This strategy was considered in view of the strong oxidative ability of RuO_4_. The protocol was successfully applied and allowed for inserting the carbonyl functionality in the position C3 of the azanorbornane moiety. The cycloadducts **2** were straight converted into the lactams **7** and from the latter the *γ*-amino acids **6** were prepared under hydrolytic conditions [36]. With these *γ*-amino acids **6** in hand we explored the reduction reactions for the planned conversion into the desired aminols of type **5**. In this paper we wish to report the mild chemoselective reduction of *γ*-lactams and *γ*-amino acids in the presence of LiAlH_4_, detailing the procedures to prepare a small library of stereodefined aminols from *N*-alkyl substituted 2-azanorbornane derivatives. An overall and complete view of the synthetic connections among different starting materials and final products is given as a definitive picture of the new chemistry of 2-azanorbornene derivatives.

## 2. Results

### 2.1. The Reduction of the Regioisomeric Isoxazoline-*γ*-amino Acids

**Scheme 2:**
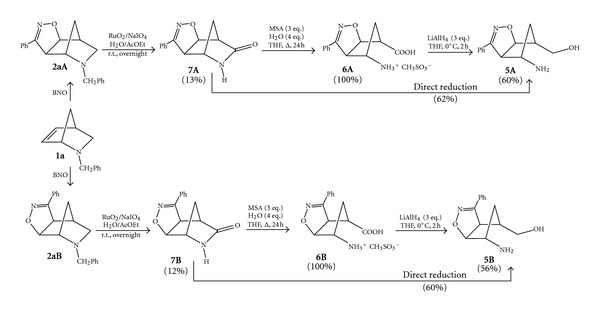


*N*-Benzyl-2-azanorborn-5-ene **1a** was prepared by addition of freshly distilled cyclopentadiene to an aqueous solution of benzylamine hydrochloride and 37% aqueous formaldehyde in an ADA reaction according to the well-known procedure [15, 16]. The 1,3-dipolar cycloaddition of BNO with **1a** was performed by generating the 1,3-dipole with the *in situ* procedure [37], affording the two regioisomeric cycloadducts **2aA** and **2aB** in 49% and 43% yields, respectively (Scheme 2) [14].

The regioisomeric cycloadducts **2aA** and **2aB** were oxidized by using the catalytic system RuO_2_/NaIO_4_ with the H_2_O/AcOEt biphasic conditions [38, 39]. The protocol needs only a catalytic amount of the expensive RuO_2_·H_2_O (10–20% mol.) in the presence of 2.5 equivalents (with respect to the substrate to be oxidized) of sodium periodate as oxidant in order to regenerate the oxidizing species RuO_4_ at the highest oxidation state after the end of the catalytic cycle [40–42]. Typically, RuO_2_·H_2_O is added to a solution of NaIO_4_ in water in inert atmosphere and left under stirring for 30 minutes. The formation of the RuO_4_ species at the highest oxidation state becomes clear because of the bright yellow colour solution.

**Scheme 3:**
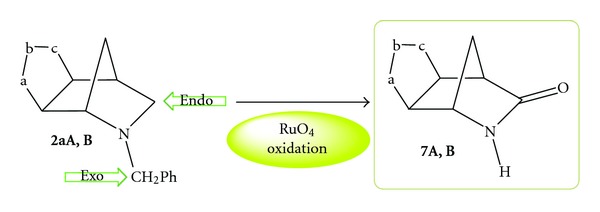


At this point, ethyl acetate solutions of the substrate are added in one portion. The reaction mixture instantly turns an opaque-black colour. The cycloadducts **2aA,B** disappear almost completely in the reaction time (TLC monitoring required to fix the end of the reaction) and the formation of three new products for each regioisomer was observed [36]. They are all amide derivatives where the oxidation occurred both on the Endocyclic and Exocyclic active methylenes adjacent to the nitrogen atom. When the oxidation on the Endocyclic methylene occurred, the process is accompanied by detachment of the benzyl group (vide infra); these are our target regioisomeric lactams of type **7A,B** and are formed in 13% and 12% yields, respectively (Scheme 3). These modest yields can be improved by 10% by adding the ethyl acetate solutions of the substrates to be oxidized portionwise.

The transformation of the lactams **7A,B** into the desired *γ*-amino acids **6A,B** was secured by the easy and quantitative hydrolysis in the presence of 3 equivalents of methanesulfonic acid (MSA) and 4 equivalents of water in THF at reflux for 24 h [43]. Insoluble methanesulfonic acid salts of the regioisomeric amino acids **6A,B** separate from the organic solution and the products are easily isolated in quantitative yields by simple filtration (Scheme 2).

The reduction of the *γ*-amino acids **6A,B** was then investigated to find the best and mildest reaction condition to prepare the desired aminols **5A,B**, where the isoxazoline ring must be preserved during the reduction process. The use of classical borane-tetrahydrofuran (BH_3_·THF) as an electrophilic agent to reduce the carboxylic acid functionality was found weakly enough to save the isoxazoline moiety but did not give the expected results [44], leaving unaltered the starting materials. An alternative and mild method to reduce the COOH group in the presence of other functionalities takes advantage of the coupling of NaBH_4_ with I_2_ but, again, the starting amino acids were recovered mostly unchanged [45]. We decided to test LiAlH_4_ to perform this reduction, in spite of the known tendency of this strong reductive agent to reduce and open isoxazole and isoxazoline rings [46]. For this reason we performed several tests on small amounts (analytical scale) of the acids **6A,B** before scaling up for synthetic purposes. We also applied different experimental conditions, finding at the end the mildest and promising, possible. Solutions in anhydrous THF of the regioisomeric *γ*-amino acids **6A,B** were treated with 3 equivalents of LiAlH_4_ added portionwise at 0°C under stirring and the reactions were monitored by TLC until disappearance of the starting compounds. In 1-2 hours the reactions were completed and excess LiAlH_4_ was rapidly destroyed by adding ethyl acetate at 0°C up to room temperature. The organic phases were then washed twice with brine and dried over anhydrous Na_2_SO_4_. Upon evaporation of the solvent, the residues furnished successfully the desired aminols **5A,B** in 60% and 56% yields, respectively, which were purified by MPLC (Scheme 2). The products were characterized and found identical to authentic samples of the aminols prepared according to the previously reported synthetic procedure [14].

### 2.2. Direct Reduction of *γ*-Lactams to Aminols

The successful and chemoselective reduction of the *γ*-amino acids **6A,B** prompted us to explore the feasibility of the direct reduction of lactams **7A,B **bypassing the intermediate step to amino acids by applying the same or a slightly modified protocol. The regioisomeric lactams **7A,B** were dissolved in anhydrous THF and 3 equivalents of LiAlH_4_ were added portionwise under stirring at 0°C. Upon monitoring by TLC, the reactions reached completion in 2 h affording, after a rapid quenching at 0°C of excess LiAlH_4_ with ethyl acetate, the desired aminols **5A,B** in 62% and 60% yields, respectively, (Scheme 2). However, the great oxidative power, LiAlH_4_ can be used successfully under mild operative conditions to selectively reduce also the amide group located in the strained structure of the tricyclic *γ*-lactams **7A,B**. For these reasons, as the nucleophilic hydride attacks the carbonyl group, the amide N–C=O single bond weakens and ring opening is favoured by the relief of strain due to the collapse of the tricyclic heterocycle into a bicyclic structure [47–50]. 

**Scheme 4:**
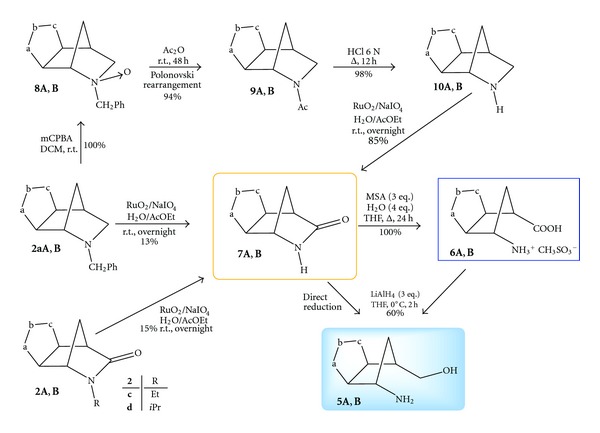


These results somewhat require to summarize the various pathways available to reach the target aminols from different sources. The synthetic connections among the 1,3-dipolar cycloadducts to the 2-azanorbornene derivatives, intermediates, and final products are reported in Scheme 4. The key lactams **7A,B** are obtainable from direct RuO_4_-mediated oxidation of *N*-benzyl cycloadducts **2aA,B** [36].

Alternatively, a more efficient and expedite route to the lactams **7A,B** takes advantage of the already applied protocol [14] through the oxidation of the regioisomeric cycloadducts **2aA,B** to the corresponding *N*-oxides **8A,B**, followed by the Polonovski rearrangement to prepare the *N*-acetyl derivatives **9A,B** in quantitative yields. A facile hydrolysis with boiling HCl 6 N for 12 h afforded quantitatively the deprotected cycloadducts **10A,B** (Scheme 4) and, by applying the oxidation protocol with RuO_2_/NaIO_4_, the latter were converted in good yields into the lactams **7A,B**. A third approach to lactams **7A,B** has been recently accounted and some regioisomeric *N*-alkyl derivatives of type **2c,d**, where a methyne or a methylene group is attached to the nitrogen atom, can be oxidized under the usual protocol into the lactams **7A,B** in modest yields [45]. Having the lactams **7A,B** in hand, the desired final targets, *γ*-amino acids **6A,B** and/or aminol **5A,B**, can be pursued through the easy application of the reported methods. The synthetic relevance of these aminols is linked to their use in the linear construction of purine and pyrimidine heterobases, that is, the synthesis of carbocyclic nucleosides with potential antiviral activity [14, 30, 31].

### 2.3. N-Alkyl Aminols

**Scheme 5:**
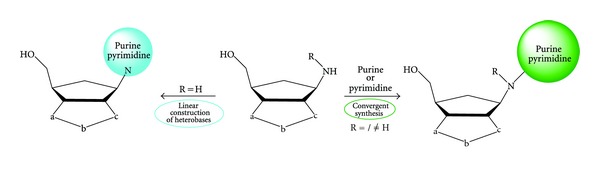


The RuO_4_-catalyzed oxidation reactions of some *N*-alkyl substituted isoxazolino-2-azanorbornane cycloadducts, obtained from the 1,3-dipolar cycloaddition reactions with benzonitrile oxide (BNO) to the corresponding *N*-alkyl substituted 2-azanorborn-5-enes, afforded a short-cut entry to valuable bridged *γ*-lactams, potentially analogues of classical *β*-lactam antibiotics [51]. In order to have further insights on scope and limitations of the mild and chemoselective reductive protocol based on the use of LiAlH_4_, we have furtherly investigated the reduction of these novel lactams with the aim to prepare a small library of *N*-alkyl substituted aminols as valuable intermediates for the convergent synthesis of novel nucleoside analogues, where the nucleophilic nitrogen atom is used to link either commercially available or properly synthesized purine and pyrimidine bases bearing an appropriate leaving group. The strategy is reported in Scheme 5 and is complementary as well as straightforward to the desired nucleoside analogues with respect to the linear construction of purine and pyrimidine heterobases, that requires more steps to the targets [14].

**Scheme 6:**
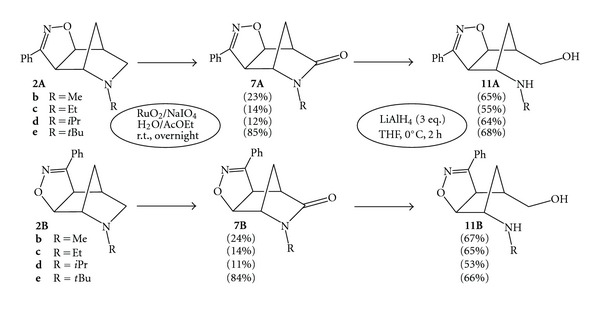


The regioisomeric cycloadducts **2A,B (b–e)** were oxidized by using the catalytic system RuO_2_/NaIO_4_ with the H_2_O/AcOEt biphasic conditions [38, 39]. The lactams **7A,B (b–e)** were isolated in moderate yields as part of a complex mixture of oxidized compounds (Scheme 6). The direct reduction reactions were conducted by dissolving the regioisomeric lactams **7A,B** in anhydrous THF and 3 equivalents of LiAlH_4_ were added portionwise under stirring at 0°C. Upon monitoring by TLC, the reactions reached completion in 1-2 h affording, after the usual workup, the desired aminols **11A,B (b–e)** from 53% to 68% yields.

The structures of the newly prepared *N*-alkyl aminols **11** rely upon their analytical and spectroscopic data. The comparison of the spectroscopic data of the aminols **11** with those of the starting lactams **7A,B** allowed for a firm assignment of the structures reported. The regioisomeric aminols **11bA,B** were clearly identified since in the ^1^H NMR spectra a new AB system appears at *δ* 3.81 for both regioisomers corresponding to the hydroxy methylene group (CH_2_–OH); moreover in the ^13^C NMR spectra the signals relative to the amide carbonyl groups at *δ* 173–176 are now missing.

The ^1^H NMR spectra of the *N*-Et substituted aminols **11cA,B** showed the hydroxy methylene groups as AB systems at *δ* 3.61 and 3.83 for compound **11cA** and at *δ* 3.59 and 3.76 for compound **11cB**; the relative ^13^C NMR spectra showed the absence of the amide carbonyl groups at *δ* 173–175. Similarly, the ^1^H NMR spectra of the *N*-*i*Pr substituted aminols **11dA,B** showed the hydroxy methylene groups as AB systems at *δ* 3.62 and 3.82 for compound **11dA** and at *δ* 3.58 and 3.81 for compound **11dB**; the relative ^13^C NMR spectra showed the absence of the amide carbonyl groups at *δ* 172–174. Finally, the ^1^H NMR spectra of the *N*-*t*Bu substituted aminols **11eA,B** showed the hydroxy methylene groups as AB systems at *δ* 3.66 for compound **11eA** and at *δ* 3.64 and 3.78 for compound **11eB**; the relative ^13^C NMR spectra showed the absence of the amide carbonyl groups at *δ* 173–175.

All the structures of the aminols **11A,B** reported are also characterized by strong IR bands of the OH group between 3300–3375 cm^−1^, which cover the NH bands of the amine group.

## 3. Discussion

**Scheme 7:**
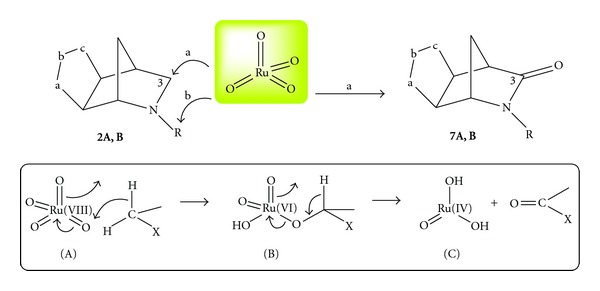


The RuO_4_-catalyzed oxidation protocol has been applied to the isoxazolino-2-azanorbornane derivatives in the search of an expedite and selective oxidation towards lactam derivatives as precursors of valuable aminols. The latter are in fact the ideal starting materials for nucleosides and natural products syntheses. It is worthwhile to remember that Ruthenium tetroxide was firstly employed in strong and quite unselective oxidations [32]. In general, the reactions required a biphasic system of organic solvent and water to be performed allowing RuO_4_ to display its complete oxidizing power [33]. Oxidation of C–H bonds represents the most intriguing property of the ruthenium tetroxide as oxidant in 1,2-dehydrogenation of alcohols and amines as well as in 1,1-dehydrogenation of saturated hydrocarbons, a quite uncommon process but extremely useful from the synthetic point of view, allowing for the introduction of oxidated functionalities on activated methylenes [38, 39]. The mechanism proposed by Bakke et al. [52–54] and widely recognised in its validity is shown in the inset of Scheme 7. The oxidation process takes place in two separate steps: (1) oxidative addition of RuO_4_ to the C–H bond (A) through a concerted transition state (TS) yielding the metal alcoholate with a reduced Ru (VI) (B); (2) fragmentation of the metal alcoholate affording the carbonyl compound while Ru (VI) is reduced at Ru (IV) (C) which must be reoxidized to Ru (VIII) by the sodium periodate to restart the catalytic cycle [33, 38, 39, 55]. A valuable alternative mechanism is the hydrogen abstraction from the C–H bond by the Ru=O fragment to afford, after ligand migration, the metal alcoholate (B) that undergoes the final oxidative step [56].

The regioselectivity of the oxidation of *N*-benzylated tertiary amines has already been presented by Petride and coworkers [34, 35]. In the case of the tricyclic 2-azanorbornane derivatives at hand, two different methylenes (*exo*cyclic and *endo*cyclic) are activated by the same nitrogen atom and the oxidation reaction proceeds with poor regioselection and both the methylenes are involved affording two different amides [36, 55]. However, when cycloadducts **2A,B** undergo oxidation by RuO_4_ on the endocyclic (path a) methylenes, the amides **7A,B** are easily obtained (Scheme 7) from moderate to fair yields.

These tricyclic amides display the intriguing competition between the amide moiety and the isoxazoline ring towards reduction in the presence of a potential variety of reductive agents. The method has been validated using LiAlH_4_ in THF as solvent at 0°C until disappearance of the starting materials in accordance with the literature findings concerning the reduction of amides under special experimental conditions [57]. The weak N–O bond of isoxazoline rings can be cleaved reductively by two principal methods: catalytic hydrogenation or treatment with LiAlH_4_ [46]. Since this ring opening is a key step in the construction of complex molecules the experimental conditions of the two reactions have been investigated thoroughly by different authors. Reductions with LiAlH_4_ are carried out predominantly in ethers as solvent (Et_2_O, THF, Dioxane) heating the solutions at reflux for several hours allowing for the preparation of the required aminol structures [46].

In the cases at hand, LiAlH_4_ is used under milder and controlled conditions at 0°C in THF calibrating the reaction time on the consume of the starting amides **7A,B** (Scheme 8), monitoring constantly by TLC the evolution of the reactions. In order to reach the complete transformation of the amides into the aminols, we used 3 equivalents of LiAlH_4_ since the reactions proceed through more than a single step (reduction, ring opening) to produce the desired aminols **5A,B** and **11A,B**, depending on the presence of an alkyl substituent on the amino group. As a consequence, the reduction conducted on the isolated amino acids **6A,B** affords the target compounds. The protocol can be nicely applied to single regioisomers of amides **7A,B** or onto the regioisomeric mixtures of the same compounds, separating the regioisomeric aminols **5A,B** and **11A,B** during the final purification through column chromatography. The results in terms of yields are quite good. Nevertheless, in some cases, in repeated experiments performed to demonstrate the reproducibility of the method, few amounts of the 2-alkyl azanorbornane cycloadducts (6–11%) were identified and isolated as a result of the amide reduction without cleavage [58–60]; that is, the chemoselective reduction of the amide group in the presence of an isoxazoline ring remains a delicate process but feasible if properly tuned up according to the structures at hand.

## 4. Conclusions

In conclusion, we have developed a short synthetic procedure for the synthesis of valuable aminols conformationally constrained because of the fused heterocyclic ring to the cyclopentane moiety with some alkyl substituents on the amino group. The key intermediates are the lactams **7A,B** whose preparation can be performed starting from the regioisomeric cycloadducts to the *N*-alkyl 2-azanorbornene **2A,B** taking advantage of the efficient catalytic oxidation by RuO_4_ [36, 51]. The chemoselective reduction of the amide groups is easily conducted in the presence of excess LiAlH_4_ under mild and controlled conditions. Concomitant ring opening afforded the desired isoxazoline-carbocyclic aminols **11A,B**, as key intermediates for our studies in nucleoside syntheses. Samples of compounds **5A,B **and **11A,B** will be used in a short-cut and linear synthesis of classical nucleosides according to the well-established protocols [14, 30, 31] as well as unusual nucleoside analogues through the convergent planned methodology.

## 5. Experimental Section

### 5.1. General

All melting points are uncorrected. Elemental analyses were done on a C. Erba 1106 elemental analyzer available in our department. IR spectra (nujol mulls or neat in case of oils) were recorded on an FT-IR Perkin-Elmer RX-1. ^1^H- and ^13^C-NMR spectra were recorded on a Bruker AVANCE 300 in the specified deuterated solvents. Chemical shifts are expressed in ppm (*δ*) from internal tetramethylsilane.

Column chromatography and tlc: silica gel 60 (0.063–0.200 mm) (Merck); eluant chloroform and chloroform/methanol 9 : 1. MPLC: Biotage FMP apparatus equipped with KP-SIL columns, eluant chloroform and chloroform/methanol 9 : 1. The identification of samples from different experiments was secured by mixed mps and superimposable IR spectra.

### 5.2. Materials

 Lactams **7A,B (a–e)** and the amino acids **6A,B** were prepared according to previously reported procedures [36, 51]. Ruthenium (IV) oxide hydrate (RuO_2_·H_2_O), sodium periodate, and LiAlH_4_ are from Sigma-Aldrich. All other reagents and solvents were purchased and used without any further purification. Anhydrous THF was distilled over metallic sodium.

### 5.3. General Procedure for Reduction Reactions with LiAlH_4_

Solutions in anhydrous THF of the regioisomeric *γ*-amino acids **6A,B** or of the regioisomeric lactams **7A,B** were treated with 3 equivalents of LiAlH_4_ added portionwise at 0°C under stirring and the reactions were monitored by TLC until disappearance of the starting compounds. In a couple of hours the reactions were completed and excess LiAlH_4_ was rapidly destroyed by adding ethyl acetate from 0°C to room temperature. The organic phases were then washed twice with brine and dried over anhydrous Na_2_SO_4_. Upon evaporation of the solvent, the residues furnished the desired aminols **11A,B**, which were purified by MPLC. Aminols **5A,B** were found identical to previously prepared samples [14]. New compounds were fully characterized as follows.

**11Ab**

(65%), pale yellow oil. IR: *ν*_max⁡_ 3370, 1569 cm^−1^. *R_f_* (chloroform/methanol 9 : 1) 0.26. ^1^H-NMR (300 MHz, CDCl_3_): *δ*_H_ 2.01 (1H, d, *J* 15 Hz, H–CH); 2.21 (1H, m, HC–H); 2.70 (1H, m, CH); 2.80 (3H, d, *J* 5 Hz, CH_3_); 3.81 (2H, d, *J* 6 Hz, C*H_2_*–OH); 4.51 (1H, d, *J* 9 Hz, H4_isox_); 4.72 (1H, d, *J* 6 Hz, CH–N); 5.26 (1H, d, *J* 9 Hz, H5_isox_); 7.54 (3H, m, arom.); 8.06 (2H, m, arom.). ^13^C-NMR (75 MHz, CDCl_3_) *δ*_C_ 30.7 (CH_2_), 32.9 (CH), 49.5 (CH–NH), 58.8 (CH_3_–NH), 64.5 (CH_2_–OH), 86.7 (CH–C=N), 89.6 (CH–O), 127.4, 128.7, 129.7 and 130.8 (arom.), 156.7 (C=N). Anal. Calcd. for C_14_H_18_N_2_O_2_ (MW = 246.30): C, 68.27; H, 7.37; N, 11.37. Found: C, 68.30; H, 7.40; N, 11.30.

**11Bb**

(67%), yellowish oil. IR: *ν*_max⁡_ 3375, 1614 cm^−1^. *R_f_* (chloroform/methanol 9 : 1) 0.23. ^1^H-NMR (300 MHz, CDCl_3_): *δ*_H_ 1.30 (2H, m, CH_2_); 2.51 (1H, m, CH); 2.76 (3H, d, *J* 5 Hz, CH_3_); 3.81 (2H, dd, *J* 6, 2 Hz, C*H_2_*–OH); 4.26 (1H, dd, *J* 9, 2 Hz, H4_isox_); 4.77 (1H, d, *J* 3 Hz, CH–N); 5.11 (1H, b, NH); 5.36 (1H, d, *J* 9 Hz, H5_isox_); 7.46 (3H, m, arom.); 7.88 (2H, m, arom.). ^13^C-NMR (75 MHz, CDCl_3_) *δ*_C_ 30.7 (CH_2_), 32.2 (CH), 47.1 (CH–NH), 54.0 (CH_3_–NH), 66.4 (CH_2_–OH), 88.0 (CH–C=N), 90.3 (CH–O), 127.3, 128.7, 129.8 and 130.6 (arom.), 158.8 (C=N). Anal. Calcd. for C_14_H_18_N_2_O_2_ (MW = 246.30): C, 68.27; H, 7.37; N, 11.37. Found: C, 68.22; H, 7.40; N, 11.32.

**11Ac**

(55%), brown oil. IR: *ν*_max⁡_ 3301, 1620 cm^−1^. *R_f_* (chloroform/methanol 9 : 1) 0.27. ^1^H-NMR (300 MHz, CDCl_3_): *δ*_H_ 1.19 (3H, t, *J* 7 Hz, CH_3_); 1.63 (1H, d, HC–H); 2.16 (1H, m, H–CH); 2.74 (2H, q, *J* 7 Hz, CH_2_); 3.34 (1H, d, *J* 6 Hz, C*H*–CH_2_–OH); 3.61 and 3.83 (2H, AB syst., CH_2_–OH); 3.70 (1H, t, *J* 6 Hz, CH–N); 3.90 (1H, d, *J* 9 Hz, H4_isox_); 5.28 (1H, d, *J* 9 Hz, H5_isox_); 7.44 (3H, m, arom.); 7.72 (2H, m, arom.). ^13^C-NMR (75 MHz, CDCl_3_) *δ*_C_ 14.8 (CH_3_), 35.5 (CH_2_), 41.5 (CH), 49.3 (CH–NH), 59.8 (CH_2_–NH), 62.6 (CH_2_–OH), 64.0 (CH–C=N), 90.7 (CH–O), 126.6, 128.8, 129.1 and 129.9 (arom.), 156.6 (C=N). Anal. Calcd. for C_15_H_20_N_2_O_2_ (MW = 260.33): C, 69.20; H, 7.74; N, 10.76. Found: C, 69.28; H, 7.79; N, 10.70.

**11Bc**

(65%), brown oil. IR: *ν*_max⁡_ 3060, 1615 cm^−1^. *R_f_* (chloroform/methanol 9 : 1) 0.24. ^1^H-NMR (300 MHz, CDCl_3_): *δ*_H_ 1.19 (3H, t, *J* 7 Hz, CH_3_); 1.63 (1H, d, HC–H); 2.15 (1H, m, H–CH); 2.59 (1H, d, *J* 6 Hz, C*H*–CH_2_–OH); 2.74 (2H, q, *J* 7 Hz, CH_2_); 3.47 (1H, d, *J* 3 Hz, CH–N); 3.59 and 3.76 (2H, AB syst., C*H_2_*–OH); 3.54 (1H, d, *J* 9 Hz, H4_isox_); 4.90 (1H, d, *J* 9 Hz, H5_isox_); 7.42 (3H, m, arom.); 7.70 (2H, m, arom.). ^13^C-NMR (75 MHz, CDCl_3_) *δ*_C_ 14.8 (CH_3_), 35.2 (CH_2_), 41.3 (CH), 46.7 (CH–NH), 53.8 (CH_2_–NH), 64.6 (CH_2_–OH), 64.9 (CH–C=N), 91.9 (CH–O), 126.6, 126.8, 128.8 and 129.9 (arom.), 159.3 (C=N). Anal. Calcd. for C_15_H_20_N_2_O_2_ (MW = 260.33): C, 69.20; H, 7.74; N, 10.76. Found: C, 69.29; H, 7.79; N, 10.70.

**11Ad**

(64%), yellowish oil. IR: *ν*_max⁡_ 3294, 1654 cm^−1^. *R_f_* (chloroform/methanol 9 : 1) 0.30. ^1^H-NMR (300 MHz, CDCl_3_): *δ*_H_ 1.14 (6H, d, *J* 6 Hz, CH_3_); 1.62 (1H, m, H–CH); 2.23 (1H, m, HC–H); 2.74 (1H, m, CH); 2.93 (1H, m, Me_2_–CH); 3.47 (1H, m, CH–N); 3.62 and 3.82 (2H, AB syst., C*H_2_*–OH); 3.85 (1H, d, *J* 9 Hz, H4_isox_); 5.28 (1H, d, *J* 9 Hz, H5_isox_); 7.44 (3H, m, arom.); 7.68 (2H, m, arom.). ^13^C-NMR (75 MHz, CDCl_3_) *δ*_C_ 22.1 (CH_3_), 23.2 (CH_3_), 35.4 (CH_2_), 45.8 (CH), 49.4 (CH–NH), 59.4 (CH–NH), 60.4 (CH_2_–OH), 64.0 (CH–C=N), 90.8 (CH–O), 126.6, 126.7, 128.8 and 129.9 (arom.), 156.7 (C=N). Anal. Calcd. for C_16_H_22_N_2_O_2_ (MW = 274.36): C, 70.04; H, 8.08; N, 10.21. Found: C, 70.00; H, 8.10; N, 10.31.

**11Bd**

(53%), yellow oil. IR: *ν*_max⁡_ 3360, 1668 cm^−1^. *R_f_* (chloroform/methanol 9 : 1) 0.27. ^1^H-NMR (300 MHz, CDCl_3_): *δ*_H_ 1.16 (6H, d, *J* 6 Hz, CH_3_); 1.60 (1H, m, H–CH); 2.18 (1H, m, HC–H); 2.56 (1H, m, CH); 2.97 (1H, m, Me_2_–CH); 3.59 (1H, m, CH–N); 3.58 and 3.81 (2H, AB syst., C*H_2_*–OH); 4.22 (1H, d, *J* 9 Hz, H4_isox_); 4.89 (1H, d, *J* 9 Hz, H5_isox_); 7.42 (3H, m, arom.); 7.70 (2H, m, arom.). ^13^C-NMR (75 MHz, CDCl_3_) *δ*_C_ 22.1 (CH_3_), 23.0 (CH_3_), 35.4 (CH_2_), 45.6 (CH), 46.8 (CH–NH), 53.8 (CH–NH), 61.4 (CH_2_–OH), 64.8 (CH–C=N), 92.1 (CH–O), 126.6, 126.7, 128.8 and 129.9 (arom.), 159.4 (C=N). Anal. Calcd. for C_16_H_22_N_2_O_2_ (MW = 274.36): C, 70.04; H, 8.08; N, 10.21. Found: C, 70.09; H, 8.11; N, 10.11.

**11Ae**

(68%), brown oil. IR: *ν*_max⁡_ 3300, 1558 cm^−1^. *R_f_* (chloroform/methanol 9 : 1) 0.28. ^1^H-NMR (300 MHz, CDCl_3_): *δ*_H_ 1.19 (9H, s, tBu); 1.76 (1H, d, *J* 14 Hz, H–CH); 2.17 (1H, m, HC–H); 2.68 (1H, d, *J* 4 Hz, CH); 3.52 (1H, d, *J* 6 Hz, CH–N); 3.66 (2H, dd, *J* 11, 3 Hz, C*H_2_*–OH); 4.00 (1H, d, *J* 9 Hz, H4_isox_); 4.38 (2H, b, OH and NH); 5.25 (1H, d, *J* 9 Hz, H5_isox_); 7.43 (3H, m, arom.); 8.70 (2H, m, arom.). ^13^C-NMR (75 MHz, CDCl_3_) *δ*_C_ 29.2 (CH_3_), 37.2 (CH_2_), 49.9 (CH), 52.2 (CH–NH), 57.2 (C–NH), 62.1 (CH_2_–OH), 63.7 (CH–C=N), 90.4 (CH–O), 125.8, 126.8, 128.0 and 129.4 (arom.), 156.3 (C=N). Anal. Calcd. for C_17_H_24_N_2_O_2_ (MW = 288.38): C, 70.80; H, 8.39; N, 9.71. Found: C, 70.88; H, 8.39; N, 9.70.

**11Be**

(66%), light brown oil. IR: *ν*_max⁡_ 3300, 1579 cm^−1^. *R_f_* (chloroform/methanol 9 : 1) 0.26. ^1^H-NMR (300 MHz, CDCl_3_): *δ*_H_ 1.25 (9H, s, tBu); 1.68 (1H, d, *J* 14 Hz, H–CH); 2.25 (1H, m, HC–H); 2.51 (1H, d, *J* 8 Hz, CH); 3.25 (2H, b, OH and NH); 3.67 (1H, d, *J* 6 Hz, CH–N); 3.64 and 3.78 (2H, AB syst., *J* 11, 3 Hz, C*H_2_*–OH); 4.17 (1H, d, *J* 9 Hz, H4_isox_); 4.85 (1H, d, *J* 9 Hz, H5_isox_); 7.43 (3H, m, arom.); 7.71 (2H, m, arom.). ^13^C-NMR (75 MHz, CDCl_3_) *δ*_C_ 29.6 (CH_3_), 38.0 (CH_2_), 47.0 (CH), 51.9 (CH–NH), 53.8 (C–NH), 59.1 (CH_2_–OH), 64.8 (CH–C=N), 94.5 (CH–O), 125.7, 126.8, 127.5, 128.5, 128.8 and 129.9 (arom.), 159.7 (C=N). Anal. Calcd. for C_17_H_24_N_2_O_2_ (MW = 288.38): C, 70.80; H, 8.39; N, 9.71. Found: C, 70.79; H, 8.42; N, 9.74.

## Figures and Tables

**Scheme 8:**